# Correlation between ground-glass opacity on pulmonary CT and the levels of inflammatory cytokines in patients with moderate-to-severe COVID-19 pneumonia

**DOI:** 10.7150/ijms.56683

**Published:** 2021-04-16

**Authors:** Zubo Wu, Xiaoping Liu, Jie Liu, Feng Zhu, Yali Liu, Yalan Liu, Hua Peng

**Affiliations:** 1 Department of Pediatrics, Union Hospital, Tongji Medical College, Huazhong University of Science and Technology, 1277 Jiefang Avenue, Wuhan, 430022, P.R. China.; 2 Department of Emergency and Pediatrics, Shenzhen Baoan Women's and Children's Hospital, Jinan University, Shenzhen, 518102, P.R.China.; 3 Clinical Center of Human Gene Research, Union Hospital, Tongji Medical College, Huazhong University of Science and Technology, 1277 Jiefang Avenue, Wuhan, 430022, P.R. China.; 4 Department of Cardiology, Tongji Medical College, Union Hospital, Huazhong University of Science and Technology, 1277 Jiefang Avenue, Wuhan, 430022, P.R. China.

**Keywords:** COVID-19, SARS-CoV-2, GGO, Moderate-to-severe, Inflammatory cytokines, Risk factors.

## Abstract

**Objectives:** Comparative analysis of laboratory data in moderate-to-severe COVID-19 patients presenting with or without ground-glass opacities (GGOs).

**Methods:** This retrospective study examined 61 patients with moderate-to-severe COVID-19, as defined by the report of the WHO-China Joint Mission on COVID-19. All patients were admitted to the Department of Infectious Diseases, Wuhan Union Hospital from Dec 28, 2019 to Feb 22, 2020 and classified into a GGO group or a non-GGO group based on CT results. The clinical characteristics and laboratory data of the two groups were compared. Data were analyzed using univariate and multivariate analysis, and using receiver operating characteristic (ROC) analysis.

**Results:** Forty-five patients were in the GGO group (73.8%, 21 females, 24 males, mean age 54.8±17.8 years) and 16 were in the non-GGO group (26.2%, 11 females, 5 males, mean age 53±14.9 years). The levels of IL-2, IL-4, and IFN-γ were greater in the GGO group (all P<0.05). ROC analysis indicated that an elevated level of IL-2 was a good predictor of GGO (area under the curve: 0.716, optimal cutoff: 3.205 pg/mL, 53.8% sensitivity, 87.5% specificity, p<0.05). Multivariate analysis showed that IL-2 level was a significant and independent risk factor for lung GGO (OR: 8.167; 95% CI: 1.63, 40.8; P<0.05).

**Conclusions:** There were correlations between GGO in the lungs of patients with moderate-to-severe COVID-19 and the levels of IL-2, IL-4, and INF-γ. IL-2 was a significant and independent risk factor for GGO. These findings provide a basis for studying the mechanism of pulmonary lesions in COVID-19 patients.

## Introduction

Researchers first reported coronavirus disease 2019 (COVID-19), which is caused by the severe acute respiratory syndrome virus 2 (SARS-CoV-2), in Wuhan during December 2019 1. SARS-CoV-2 is a single-stranded positive-sense RNA virus that is in the *Nidovirales* order, the *Coronaviridae* family, and the *Betacoronavirus* genus 2. Similar to diseases caused by related coronaviruses, SARS-CoV-1 and Middle East respiratory syndrome-related coronavirus (MERS-CoV), COVID-19 can lead to severe respiratory symptoms, but SARS-CoV-2 has much greater infectivity than these other viruses. COVID-19 is now widespread in North America and Europe.

There are currently no effective treatments for patients with COVID-19 1, 3, 4. The primary characteristics of COVID-19 from computed tomography (CT) imaging are bilateral pulmonary ground-glass opacity (GGO), “crazy paving” pattern, airway changes, and reversed halo sign. There may also be other manifestations, such as consolidation and interlobular septal thickening. GGO is the earliest abnormality to appear and is also the major pulmonary change 5. A previous study reported that 73 of 81 COVID-19 patients (80%) had GGOs 6 and another study reported that 98% of COVID-19 patients had GGOs 7. A study of 1099 patients with acute respiratory disease (ARD) due to COVID-19 reported that 76.4% of these patients had abnormal chest CT image results, and that ground-glass opacity (GGO) was the most common abnormality 8.

As COVID-19 worsens, an individual GGO may increase and merge with other GGOs, and then evolve into fibrous streaks and solid nodules; alternatively, the resolution of GGOs may be related to improved patient condition 9, 10. A GGO on pulmonary CT manifests as a cloudy light-shadow nodule with slightly increased density and a fan-shaped distribution of pulmonary segments or lobes. An air bronchogram may be present in sheet-like shadows, or the consolidation may be accompanied by signs of bronchial ventilation 11. A recent study of the postmortem biopsy of a COVID-19 patient identified pulmonary edema and hyaline membrane formation, and these are considered the potential pathological driving forces of the GGO 12. However, the pathogenesis of GGO in the lungs of patients with moderate or severe COVID-19 is still unclear.

In viral pneumonia, the abnormal release of proinflammatory cytokines leads to apoptosis of lung epithelial and endothelial cells, thus destroying the pulmonary microvascular and alveolar epithelial cell barrier, leading to vascular leakage, alveolar edema, and hypoxia 13. COVID-19 is characterized by an excessive inflammatory response and the release of a large number of pro-inflammatory cytokines. Several studies found that this cytokine storm is directly related to lung injury, multiple organ failure, and poor prognosis in patients with severe COVID-19^14^, ^15^. ARDS with hypoxic saturation is the main cause of death from COVID-19. Although the exact mechanism by which COVID-19 causes ARDS is not fully clear, excessive production of pro-inflammatory cytokines is considered to be one of the main factors 10, 16.

In this study, we examined the independent risk factors for GGO in the lungs of patients with moderate or severe COVID-19.

## Materials and Methods

This study was reviewed and approved by the Institutional Review Board of Union Hospital, Huazhong University of Science & Technology (Wuhan, China). The local Ethics Committee approved this study and each patient provided informed consent.

### Patients

This single center, retrospective case-control study analyzed patients who had moderate or severe COVID-19 pneumonia and were diagnosed in our hospital between Dec 28, 2019 and Feb 22, 2020. The diagnostic criteria for moderate and severe pneumonia due to COVID-19 are based on the report of the WHO China Joint Investigation Mission on COVID-19 17. Moderate disease was defined as laboratory confirmed SARS-CoV-2 infection with pneumonia. Severe disease was defined by laboratory confirmed SARS-CoV-2 infection with dyspnea, respiratory rate greater than 30 per min, blood oxygen saturation (SpO_2_) of 93% or less, PaO2/FiO_2_ ratio below 300, and/or lung infiltration in more than 50% of the lung field within 24 to 48 h. Patients with critical disease (respiratory failure requiring mechanical ventilation, septic shock, and/or organ failure requiring intensive care) were not included 17. The inclusion criteria were: throat swab tested using RT-PCR and confirmed as positive for SARS-CoV-2; complete admission information and clinical data; and moderate or severe COVID-19. The exclusion criteria were: missing or incomplete data; immunologic deficiency or autoimmune disease; excessive use of a corticosteroid or immunosuppressant within 1 month before admission; and pneumonectomy and/or CT motion artifacts. Based on initial chest CT results at admission, the 61 patients were classified into a GGO group (n = 45) or a non-GGO group (n = 16). The clinical and laboratory data of these two groups were compared.

### Acquisition and analysis of lung CT images

All included patients received 64-slice CT scans of their lungs on the first day of admission using a SOMATOM Definition AS+ (Siemens Healthineers, Forchheim, Germany). The lung CT scan ranged from the apex to the bottom. Each lung CT result was interpreted by a radiologist with more than 10 years experience. The CT scanning parameters were as follows: tube voltage = 120 kVp, pitch = 1.2, matrix = 512 × 512, field of view = 350 mm × 350 mm, and with automatic tube current modulation. All images were then reconstructed with a slice thickness of 1.5 mm at the same increment 18, 19. After each patient completed the examination, the testing instrument and the testing room were strictly disinfected with an air sterilizer and 75% ethanol. The determination GGO of the lungs was in strict accordance with the literature 5.

### Treatments

All patients were hospitalized and received isolation and treatment in accordance with the therapeutic principles based on the 2019-nCoV guidelines (Trial Version 5) proposed by the National Health Commission of China 20.

### Data collection

Patient demographic data (gender, age, etc.), presence of fever, blood indices (hemoglobin level and counts of white blood cells, neutrophils, lymphocytes, monocytes, and platelets), C-reactive protein (CRP), and immune-related indices (IL-2, -4, -6, and -10; TNF-α, IFN-γ; CD3+, CD3+CD4+, and CD3+CD8+ T cells) were recorded. The presence of comorbidities (hypertension, diabetes, and cerebrovascular diseases) was also recorded.

### Statistical analysis

SPSS version 19.0 software was used for statistical analysis. Continuous variables with normal distributions are presented as means ± standard deviations (SDs), and those with non-normal distributions as medians and interquartile ranges (IQRs). Count data are presented as numbers and percentages. Comparisons of categorical variables were performed using the chi-square test or using Fisher's exact test if the expected count of at least one cell was less than 5. Comparisons of continuous variables were performed using the *t*-test (if normally distributed) or the Mann-Whitney U test (if not normally distributed). For receiver operating characteristic (ROC) analysis, the optimal cutoff value was the point closest to the upper-left corner of the ROC curve. A multiple logistic regression model was established using categorical data to determine the potential risk factors for GGO by comparing patients with moderate and severe disease. A P value below 0.05 was considered significant.

## Results

### Demographic and clinical features of the patients

We examined 61 patients with moderate or severe COVID-19 during a 2 month period. Based on the CT results, there were 45 patients (73.8%) with GGOs group and 16 patients (26.2%) without GGOs (Table 1). These two groups had similar mean age (54.8 ± 17.8 *vs.* 53 ± 14.9 years, P = 0.716) and similar percentages of males and females (P = 0.129). Hypertension and diabetes were the most common comorbidities, but the two groups did not differ significantly in comorbidities. Among all 61 cases, 5 patients (8.2%) had severe disease, all of whom were in the GGO group.

### Univariate analyses

We analyzed 16 different laboratory parameters (Table 2). Univariate analysis showed that age, gender, fever, WBC count, hemoglobin, neutrophil count, lymphocyte count, monocyte count, platelet count, CRP, IL-6, IL-10, TNF-α, CD3+ T cells (%), CD3+CD8+T cells (%), and CD3+CD4+T cells (%) were not significantly different between the GGO and non-GGO groups (all P > 0.05). However, univariate analyses showed that the GGO group had significantly higher levels of IL-2, IL-4, and INF-γ (all P < 0.05).

### ROC analysis

**Figure 1:**
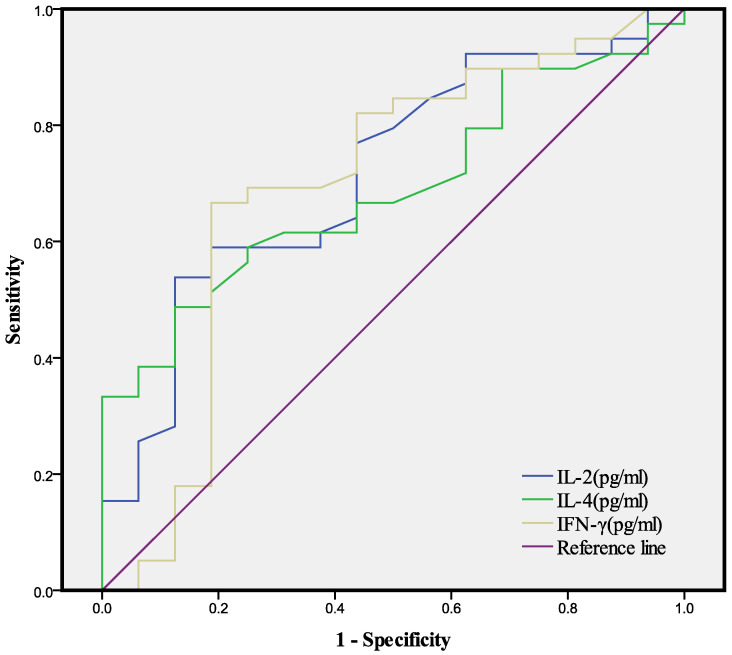
ROC analysis of IL-2, IL-4, and IFN-γ for the determination of GGO (see Table 3).

We then used each of the 3 indices that were significantly different between the two groups (IL-2, IL-4, IFN-γ) for ROC analysis, with calculations of the area under the curve and optimal cutoff value for each index (Table 3, Fig. 1). The AUC was 0.716 for IL-2, 0.789 for IL-4, and 0.696 for IFN-γ and the optimal cutoff value was 3.205 pg/mL for IL-2, 3.175 pg/mL for IL-4, and 1.875 pg/mL for IFN-γ.

### Conversion of continuous variables into bi-categorical variables and multivariate logistic regression

We converted each of the 3 indices (IL-2, IL-4, and IFN-γ) into a bi-categorical variable based on its optimal cutoff value, and then performed multivariable analysis to determine the relationship of each index with GGO (Table 4). The results showed that an IL-2 level above 3.205 was significantly and independently associated with an increased risk of pulmonary GGO (OR: 8.167; 95% CI: 1.63, 40.8; P < 0.05), but there were no significant associations of IL-2 or IFN-γ with pulmonary GGO (both P > 0.05).

## Discussion

The present retrospective case-control study of 61 patients with moderate or severe COVID-19 compared 45 patients who had GGOs in the lungs with 16 patients who had no GGOs. Our analysis of clinical and other data collected at admission indicated that the levels of three cytokines — IL-2, IL-4, and IFN-γ — had positive correlations with the presence of GGOs. Our subsequent multivariate logistic regression analysis showed that IL-2 was a significant and independent risk factor for GGOs patients with moderate or severe COVID-19.

GGO in a CT image is defined by the presence of increased pulmonary opacity and preservation of the bronchial and vascular margins, different from the increased homogeneity of pulmonary consolidations and burr signs in blood vessels and airway walls 11. GGO is a common and nonspecific finding in chest X-rays and CT, and occurs in patients with many pulmonary diseases 21. Pneumonia and other diseases are characterized by lung damage in which the inflammatory cells suffuse alveolar spaces and walls, and pulmonary interstitial thickening leads to an increased density relative to air, manifesting as GGO in a CT image 22. The radiographic imaging results of patients with SARS-CoV-2 are variable, and there may be pulmonary consolidation, subsegmental pulmonary vasodilation, interlobular septa thickening, with or without air bronchogram, but GGO is the most common finding from pulmonary CT 23. Although GGO cannot be considered pneumonia-specific for COVID-19 in CT qualitative analysis, quantitative analysis of GGOs and fibrosis alterations on chest CT can identify COVID-19 patients 24.

Previous research 25 showed that the CT abnormalities in patients with COVID-19 pneumonia were more obvious than in those with non-COVID-19 pneumonia, in that the COVID-19 group had a higher frequency of GGO (91% *vs.* 68%, P < 0.001). Another study 26 compared patients hospitalized with acute respiratory distress syndrome (ARDS) caused by COVID-19 or H1N1 and also found that the lung CTs of the COVID-19 patients had a higher frequency of GGO (94.5% *vs.* 45.3%). SARS-CoV-2 infection induces an excessive and prolonged cytokine/chemokine response in some individuals — a cytokine storm. An uncontrolled cytokine storm can lead to ARDS or multiple organ dysfunction (MOD), and ultimately to physiological deterioration and death 27. Other studies of COVID-19 patients reported that their CT images had a characteristic GGO, indicating that the lungs contained fluid 28. A recent case report, which described the autopsy of a patient with COVID-19, confirmed that their lungs were filled with a transparent liquid jelly-like substance, similar to that in the lungs of drowning victims 12. Although the nature of this substance has yet to be determined, a study of COVID-19 patients showed it was due to the cytokine storm that promoted pneumonia and pulmonary GGO 29. At present, the relationship of the cytokine storm with the progression of COVID-19 and how it causes GGO of the lungs is still unclear. This remains an important topic that requires additional research.

We examined 61 patients with moderate-to-severe COVID-19 in this study. CT of their lungs showed that 73.8% of them had GGO, consistent with the findings of previous studies. We found that the lung CTs of patients with moderate or severe pneumonia due to COVID-19 were more likely to have GGO if they had increased levels of IL-2, IL-4, and INF-γ, and that IL-2 was significantly and independently associated with GGO. These results are consistent with previous research which reported that the cytokine storm in patients with COVID-19 pneumonia was associated with increased levels of IL-2 and INF-γ 30. Other researchers also reported elevated levels of IL-2, IL-4, and INF-γ in patients with COVID-19 31, 32, and that these levels were highly correlated with disease progression. In addition, critically ill COVID-19 patients had significantly higher levels of IL-2, IL-4, and INF-γ than those with moderate or severe disease 33, 34, and those with severe COVID-19 had higher levels of certain cytokines than those with mild disease 32. This confirms that the severity of the cytokine storm is related to disease severity 32. In support of this interpretation, a previous study of COVID-19 patients showed that intravenous injection of mesenchymal stem cells (MSCs) reduced the pulmonary GGO and the infiltration of inflammatory cells in the lungs by inhibiting the cytokine storm and by promoting tissue repair and regeneration 16. Based on our results and these previous studies, we speculate that IL-2, IL-4, and INF-γ are involved in the infiltration of alveolar inflammatory cells and lung injury caused by COVID-19. Although our GGO group had greater levels of IL-2, IL-4, and INF-γ than the non-GGO group, the mechanism by which these inflammatory factors cause ARDS and lung injury is still unclear.

A total of 86.9% of our patients had significantly elevated IL-6 levels, consistent with previous reports 35. An elevated serum IL-6 level is common in patients with severe COVID-19 34. Chen et al. 36 reported that the serum SARS-CoV-2 viral load in critically ill patients was highly correlated with the serum IL-6 level (R = 0.902). High levels of IL-6 may also cause an increase in neutrophils and a decrease in lymphocytes 37. Moreover, IL-6 may promote the development of ARDS in COVID-19 patients, and an increased IL-6 level may be a sign of more aggravated disease 37. In contrast, we found no statistical difference in the IL-6 levels in our GGO and non-GGO groups. The reason for this discrepancy may that we only examined patients with moderate or severe disease, and excluded critically ill patients. Thus, we are planning follow-up clinical studies that have larger sample sizes to compare the data of patients with different disease severities and clarify the mechanism of the formation of lung GGOs in patients with COVID-19.

This study has certain limitations. In particular, this was a retrospective single-center study and the number of cases was relatively small. A large multi-center study would provide more reliable results. In addition, we did not examine patients with other viral infections. Analysis of the relationship between lung CT findings and cytokine levels of patients with other viral infections may help us distinguish COVID-19 pneumonia from other lung infections. We also did not study the relationship between CT results and the levels of cytokines during the initial stage of infection nor in patients with asymptomatic SARS-CoV-2 infections. We believe that further studies of the relationship between changes in CT images and the levels of cytokines may help to monitor and predict the course of disease, and may also provide support for clinical decision-making.

## Conclusion

We propose that the appearance of GGOs in the CT images of patients with moderate-to-severe COVID-19 is related to increased levels of certain cytokines, and that IL-4 and INF-γ play key roles in causing the cytokine storm and GGOs in these patients. Moreover, we found that IL-2 was an independent risk factor for lung GGOs in patients with moderate-to-severe COVID-19. This suggests a possible new approach for studying the mechanism of lung disease in COVID-19.

## Figures and Tables

**Table 1:** Demographic and clinical characteristics of all 61 COVID-19 patients and of those in the GGO and non-GGO groups.*

Characteristic	Total (n=61)	GGO (n=45)	nGGO(n=16)	*P* value
**Demographics**				
Age (years)		54.8±17.8	53±14.9	0.716^a^
**Gender**				
Female	32 (52.5%)	21 (46.7%)	11 (68.7%)	0.129
Male	29 (47.5%)	24 (53.3%)	5 (31.3%)	
**Comorbidities**				
Fever	40 (65.6%)	29 (72.5%)	11 (68.8%)	0.756^b^
Any additional disease	45 (73.7%)	35 (57.3%)	10 (16.3%)	0.102^b^
COPD	4 (6.6%)	2 (4.4%)	2 (12.5%)	0.267
Cardiovascular disease	3 (4.9%)	3 (6.7%)	0	0.294
Cerebrovascular disease	2 (3.3%)	2 (4.4%)	0	0.395
Chronic liver disease	2 (3.3%)	1 (2.2%)	1 (6.3%)	0.441
Diabetes	11 (18.0%)	8 (17.8%)	3 (18.8%)	0.931
Hypertension	21 (34.4%)	16 (35.6%)	5 (31.3%)	0.756
Other	3 (4.9%)	3 (6.7%)	0	0.294
**Severe disease**	5 (8.2%)	5 (11.1%)	0	0.168

* Categorical data are presented as n (%) and continuous data as mean ± SD. Variables with normal distributions were compared using the Shapiro-Wilk test, and other variables using Fisher's exact test. a, independent variables *t*-test; b, Chi-Square test.

**Table 2:** Univariate analysis of the GGO and non-GGO groups.*

Variable	GGO (n=45)	nGGO (n=16)	*P* value
WBC (×10^9^/L)	5.37 (4.26-6.67)	4.83 (3.9-7.49)	0.828
Hb (g/L)	124.6±13.9	121.5±14.1	0.453^a^
PLT (×10^9^/L)	225.2±76.7	233.7±93.3	0.723^a^
N (×10^9^/L )	3.26 (2.43-4.47)	2.85 (1.83-5.19)	0.713
L (×10^9^/L)	1.27±0.5	1.53±0.67	0.122^a^
M (×10^9^/L)	0.49 (0.38-0.61)	0.57 (0.36-0.70)	0.238
CRP (mg/L)	6.91 (2.6-29.85)	6.80 (3.14-38.2)	0.839
IL-2 (pg/mL)	3.3 (2.6-4.23)	2.55 (2.33-2.94)	**0.012**
IL-4 (pg/mL)	2.93 (2.11-4.47)	2.24 (1.63-2.89)	**0.029**
IL-6 (pg/mL)	8.83 (5.02-20.34)	5.70 (3.69-26.86)	0.258
IL-10 (pg/mL)	4.58 (3.86-5.54)	4.27 (2.91-4.64)	0.081
TNF-α (pg/mL)	3.11 (2.13-4.38)	2.37 (1.93-3.67)	0.194
IFN-γ (pg/mL)	3.02 (2.25-3.88)	2.06 (1.75-2.47)	**0.023**
CD3+CD8+T (%)	25.1±9.2	36.3±49.7	0.183^a^
CD3+ T (%)	76.44 (72.34-80.2)	79.9 (70.4-81.87)	0.726
CD3+CD4+T (%)	46.54 (41.28-51.5)	48.2 (41.06-54.28)	0.705

*Data are given as mean ± SD or median (IQR). WBC, white blood cells; Hb, hemoglobin; PLT, platelets; N, neutrophils; L, lymphocytes; M, monocytes. Gender was analyzed using the Pearson Chi-square test; Hb, PLT, N, L, and CD3+CD8+ T cells using the Welch *t* test; and other variables using the Mann-Whitney U-rank sum test. a, independent variable *t*-test; all others, Mann-Whitney U test.

**Table 3:** AUCs and optimal cutoff values of different indices from ROC analyses of IL-2, IL-4, and IFN-γ for the determination of GGO (see Figure 1).

Variable	AUC	95%CI	Sensitivity	Specificity	Optimal cutoff (pg/mL)	*P* value
IL-2	0.716	0.569-0.864	0.538	0.875	3.205	**0.012**
IL-4	0.689	0.549-0.829	0.487	0.875	3.175	**0.029**
IFN-γ	0.696	0.525-0.868	0.897	0.375	1.875	**0.023**

AUC: area under the curve; CI: confidence interval.

**Table 4:** Multivariate logistic regression analysis of 3 bi-categorical variables for the determination of GGO.

Variable	Cutoff value	OR (95%CI)	P-value
IL-2 (pg/mL)	>3.205	8.167 (1.633- 40.848)	0.011
IL-4 (pg/mL)	>1.875	2.625 (1.106-32.537)	0.218
IFN-γ (pg/mL)	>3.175	3.864 (0.489-30.531)	0.200

OR: odds ratio; CI: confidence interval

## Abbreviations

COVID-19: coronavirus disease 2019
GGO: Ground-glass opacity
SARS: severe acute respiratory syndrome
MERS: Middle East respiratory syndrome
CT: computed tomography
ARDS: acute respiratory distress syndrome
WBC: white blood cell
Hb: hemoglobin
PLT: platelet
N: neutrophil
L: lymphocyte
M: monocyte
CRP: C-reactive protein
IL-2: Interleukin-2
IL-4: Interleukin-4
IL-6: Interleukin-6
IL-10: Interleukin-10
TNF-α: Tumor necrosis factor-α
INF-γ: Interferon-γ
ROC: receiver operating characteristic
AUC: area under the curve
